# Efficacy and Safety of a Chinese Herbal Formula (Invigorating Kidney and Strengthening Spleen) in Chronic Hepatitis B Virus Carrier: Results from a Multicenter, Randomized, Double-Blind, and Placebo-Controlled Trial

**DOI:** 10.1155/2013/961926

**Published:** 2013-07-10

**Authors:** Jinsong He, Daqiao Zhou, Guangdong Tong, Yufeng Xing, Yingjie Chen, Xiaohui Zhang, Bolin Zhan, Hui Gao, Xiaozhou Zhou, Yiqun Xiong, Xinliang Liu, Lisheng Peng, Mei Qiu, Yingjun Zheng

**Affiliations:** ^1^Department of Hepatology, National Key Clinical Specialty of Liver Disease of Traditional Chinese Medicine, Shenzhen Hospital Affiliated to Guangzhou University of Chinese Medicine, No. 1 Fuhua Road, Futian District, Shenzhen 518033, China; ^2^Institute of Liver Disease, Shenzhen Hospital Affiliated to Guangzhou University of Chinese Medicine, Shenzhen 518033, China; ^3^Central Laboratory of Liver Disease, Shenzhen Hospital Affiliated to Guangzhou University of Chinese Medicine, Shenzhen 518033, China

## Abstract

A Chinese Herbal Formula (CHF) has acquired a certain therapeutic effect on chronic HBV infection. To assess the efficacy and safety of CHF on HBV replication in chronic HBV carriers, we performed a randomized, double-blind, and placebo-controlled trial involving patients from 16 centers. A total of 300 confirmed chronic HBV carriers were randomized at baseline in a ratio of 2 : 1 to receive either CHF or placebo for 52 weeks. The results showed that a greater proportion of CHF than placebo treated patients achieved virological response at week 52; the mean decline of serum HBsAg levels in the CHF group dropped more obviously than that in the control group at all stages of the treatment; however, the rates of HBeAg loss and seroconversion had no difference between the two groups. Meanwhile, were presented significant increases in IFN-**γ**; IL-2 levels and reductions in IL-4 and IL-10 levels in the treatment group compared to the control group at week 52. There were no drug-related serious adverse events. In conclusion, the treatment with 52-week CHF is safe and effective in inhibiting HBV replication in chronic HBV carriers. The ability of the compound to modulate host immune function probably contributed to this effect.

## 1. Introduction

Hepatitis B virus (HBV) infection continues to be prevalent worldwide. It was reported by the World Health Organization (WHO) that an estimated two billion people have been infected with HBV all over the world, and of these, some 350 million develop chronic infection [1]. About one million people die every year due to liver failure, cirrhosis, and hepatocellular carcinoma related to HBV infection [2]. HBV infection is endemic in China, with about 93 million people who are chronically infected. In 2006, a national survey showed a HBsAg seroprevalence of 7.18% among Chinese aged 1–59 years [3, 4].

Chronic HBV infection is a dynamic process. The natural history of prenatally or early childhood-acquired chronic HBV infection can be divided into four different phases: immune tolerant phase, immune clearance phase, residual inactive phase, and HBV reactivation phase [5]. Patients in the immune tolerant phase are usually seropositive for HBeAg and have high viral loads (>2 × 10^6^ IU/mL) but with a normal serum alanine aminotransferase (ALT) and normal or nearly normal findings on liver histology [6]. The latter are termed asymptomatic chronic HBV carriers. In this phase, the spontaneous HBeAg loss is very low, and these patients are highly contagious because they maintain high levels of viremia. Recently, a few studies compared the HBV DNA levels in blood to the progression of chronic HBV infection. A prospective cohort study with an 11-year followup in China indicated that viral load was associated with increased mortality from hepatocellular carcinoma (HCC) and chronic liver disease among HBV carriers [7]. The REVEAL-HBV study showed that elevated serum HBV DNA level (≥10,000 copies/mL) was a strong risk predictor of HCC independent of HBeAg, serum ALT level, and liver cirrhosis. A similar association between HBV DNA levels and development of cirrhosis has also been disclosed [8, 9]. Thus, it is possible that HBV carriers in immune tolerant phase, characterized with high level of HBV DNA replication and normal or low ALT level, still have high infectivity and the risk of progress to cirrhosis and HCC.

At present, interferon (IFN) and nucleosides/nucleotides (NAs) are well accepted effective drugs available for the treatment of chronic hepatitis B. However, these two kinds of drugs have definite indications for treatment. When HBV carriers are in the immune tolerant phase, the three authoritative consensus guidelines [10–12] all recommended that treatment should not be initiated unless apparent inflammation, necrosis, and/or fibrosis are found by liver biopsy. These patients were required to be followed up at least every 3–6 months because of poor efficacy treated by IFN and NAs. But realistically, that is only an unpalatable choice, and to reduce the high risk of progress to cirrhosis and HCC, billions of chronic HBV carriers in China are seeking safe and effective treatments to suppress HBV DNA replication and associated liver disease long term. A self-administered questionnaire survey showed that one-third of patients with chronic hepatitis B had used traditional Chinese medicine (TCM) in Hong Kong [13]. A review by Stickel and Schuppan claimed that several recent surveys from Europe and the United States have demonstrated that up to 65% of patients with liver disease took herbal preparations [14].

After years of practice, TCM has acquired a certain therapeutic advance in the treatment of chronic HBV infection. Several studies in vitro indicated that Phyllanthus species could suppress the replication of HBV by inhibiting DNA polymerase activity and mRNA transcription [15–17]. In a preliminary study, the researchers found that Phyllanthus amarus treatment had a distinct curative effect (HBsAg clearance) in chronic carriers of hepatitis B virus [18]. The recent study suggested that the aqueous extracts of *Ardisia chinensis* and *Pithecellobium clypearia* have strong anti-DHBV activities in vitro, which were comparable to those of 2′, 3′-dideoxycytidine [19]. Meanwhile, several systematic reviews of Chinese medicinal herbs for chronic hepatitis B indicated that herbal compounds Fuzheng Jiedu Tang, Jianpi Wenshen recipe, and *Polyporus umbellatus* polysaccharide showed anti-HBV activities [20–22]. A recently updated systematic review of randomized controlled trials (RCTs) of TCM formulations against HBV in China (from 1998 to 2008) suggests that TCMs have a similar antiviral activity as IFN/LAM (Lamivudine) on chronic hepatitis B, which was evidenced by the loss of serum HBeAg and HBV DNA [23]. However, the early trials were flawed in terms of experimental design, and results obtained only weakly supported the utility of the TCM. By contrast, our empirical Chinese herbal formula (invigorating kidney and strengthening spleen) appears to be effective in inhibiting HBV DNA replication upon treatment of chronic HBV carrier [24]. Therefore, the aim of this study was designed to evaluate the antiviral efficacy and safety of this formula in chronic HBV carriers by a prospective, multicenter, double-blinded, placebo-controlled, and large registration trial.

## 2. Patients and Methods

### 2.1. Patients

The study was performed in 16 major hospitals from China, which belong to one of four areas: East China (Shanghai Shuguang hospital, Shanghai University of Traditional Chinese Medicine; Changhai Hospital, Second Military Medical University; The Second Hospital Affiliated to Zhejiang University of Traditional Chinese Medicine; Tai'An Hospital of Traditional Chinese Medicine), South China (Shenzhen Hospital affiliated to Guangzhou University of Chinese Medicine; Foshan Hospital of Traditional Chinese Medicine; The Third People's Hospital of Shenzhen; The Third Affiliated Hospital of Sun Yat-sen University; Guangdong Hospital of Traditional Chinese Medicine), West China (Attached Hospital of Chengdu University of Traditional Chinese Medicine; West China Hospital, West China School of Medicine, Sichuan University), and North China (Beijing Ditan Hospital, Capital Medical University; Xiyuan Hospital, China Academy of Traditional Chinese Medicine; 302 Military Hospital of China; First Hospital Affiliated to Tianjin University of Traditional Chinese Medicine; Beijing YouAn Hospital, Capital Medical University). Eligible patients comprised males and females infected by HBV, with clinical diagnosis confirming to chronic hepatitis B carrier. Diagnostic criteria were detectable hepatitis B surface antigen (HBsAg) and HBeAg in serum for more than 6 months, serum HBV DNA level greater than 20,000 IU/mL, persistently normal serum ALT, and aspartate aminotransferase (AST) levels continuous followup more than 3 times in one year and minimal or mild inflammatory histologic changes. Inclusion and exclusion criteria used for patient selection are shown in Table 1.

The study was approved by the Ethics Committees at Shenzhen Hospital affiliated to Guangzhou University of Chinese Medicine and was conducted in accordance with ethical guidelines of the 1975 Declaration of Helsinki and the Good Clinical Practice Guidelines. All enrolled subjects gave witnessed written informed consent before enrollment. The clinical trial registration identifier is ChiCTR-TRC-09000425.

### 2.2. Preparation of Medication

Chinese Herbal Formula (invigorating kidney and spleen decoction) was composed of *Epimedium*, *Cuscuta*, *Eucommia*, *Astragalus membranaceus*, *Phyllanthus urinaria*, Salvia *miltiorrhiza*, *Panax notoginseng*, and so forth, which are listed in Table 2. The placebo was composed of water soluble starch, glucosum anhydricum, edible chocolate brown pigment, and lyochromes. Both of them were made into drug granules in Shenzhen Sanjiu Medical & Pharmaceutical Co., Ltd., China, as a renowned GMP certified state-level manufacturer of concentrated herbal extracts (its products can be purchased in China). The whole production process, from validating the raw materials to the final products, strictly complied with the standards of Good Manufactory Practice and Chinese Pharmacopoeia [25]. Decoction and extraction of each dried medicinal herb was performed in a single batch, respectively. After extraction, herbal preparation was separated, concentrated, and spray-dried into the form of a granule. The chemical compositions of the final products were analyzed, while all the herbals were tested to ensure the safety for human consumption including heavy metals, microorganism contamination, and insecticides. Finally all kinds of granules were mixed in accordance with their proportion in the Chinese Herbal Formula and packed in sealed plastic sachets. The composition of a sachet of granules (38.6 g) was the same as that of 210 g raw herbs, which was daily dose of each patient. The placebo is similar to the herbal granules in shape, color, taste, and packaging. 

### 2.3. Study Design

The study was a multicenter, randomized, double-blinded, and placebo-controlled clinical trial of Chinese Herbal Formula versus placebo in a ratio of 2 : 1 for 52 weeks. Each patient was instructed to have a sachet of granules (38.6 g, either study drug or placebo), dissolved with 200 mL warm water in a cup, and to take 100 mL of the solution in the morning and the rest in the afternoon every day.

Randomization was performed within one month after the screening had been completed, using voice interactive random assortment system carried out by a third party who was not involved in patient management. After the first clinic visit at baseline, patients were required to return during weeks 4 and 12, and then every 12 weeks thereafter through week 52. At each clinic visit, laboratory tests were performed to evaluate liver function and determine the safety of treatment and possible adverse events. Serum was assayed for HBV DNA, HBsAg and antibody to HBsAg (anti-HBs), and HBeAg and antibody to HBeAg at baseline and at weeks 24 and 52. Serum helper T cell 1 and helper T cell 2 (Th1/Th2) cytokines levels, including Interleukin-2 (IL-2), Interleukin-4 (IL-4), Interleukin-10 (IL-10), and Interferon-*γ* (IFN-*γ*), were detected at baseline and at week 52. 

Patients were withdrawn from the study for any of the following reasons: elevated serum ALT (>2 × ULN, upper limit of normal), occurrence of intolerable or worsening adverse events, and failure to comply with the protocol or withdrew consent.

### 2.4. Laboratory Assays

Virus was uniformly assayed at the central laboratory of liver disease, Shenzhen Hospital affiliated to Guangzhou University of Chinese Medicine, China. Serum HBV DNA levels were quantified using the COBAS Taqman assay (Roche Diagnostics, Branchburg, NJ, USA) that has a lower limit of detection of 12 IU/mL. HBsAg, anti-HBsAg, HBeAg, and anti-HBeAg were measured by the Architect i2000 assay (Abbott Laboratories); HBsAg titer in serum was quantified according to the manufacturer's instructions. An initial manual dilution of 1 : 100 was performed on all samples. Samples with HBsAg titers of greater than 250 IU/mL were manually diluted at 1 : 500 to bring the reading within the linear range. Samples with HBsAg levels of less than 0.05 IU/mL at 1 : 100 dilution were retested undiluted. Serum cytokine levels of IL-2, IL-4, IL-10, and IFN-*γ* were detected by ELISA Kits (Pharmingen, San Diego, CA, USA) according to the manufacturer's instruction. 

### 2.5. End Points

Primary efficacy end point was the proportion of patients with a virological response at week 52. Virological response was defined as a decrease in HBV DNA levels by at least 2log_10_ units in the serum following treatment compared to pretreatment levels. The degree of decrease in HBV DNA level was assessed by log reduction at the end of treatment. 

Secondary efficacy end points were the proportion of patients with a virological response at week 24, the proportions of patients with serum HBV DNA level decreasing to less than 5log_10_ IU/mL at week 52, and mean degree of decrease in HBV DNA level at weeks 24 and 52. The mean decrease in serum HBV DNA levels was also compared among patients with different baseline HBV DNA levels to determine whether the patients with lower baseline HBV DNA levels showed a larger decrease after treatment, whether these groups differed in the frequency of HBeAg loss or seroconversion to anti-HBe, whether there was a difference in the decrease in HBsAg level (≥0.5log_10_ IU/mL from baseline) at weeks 24 and 52. Further, the mean serum levels of IL-2, IL-4, IL-10, and IFN-*γ* at week 52 were compared with pretreatment levels, as well as compared between the two groups. In addition, adverse events including symptoms, signs, and clinical laboratory abnormalities within 52 weeks was documented, and discontinuation of therapy were recorded.

### 2.6. Sample Size Determination

Chronic hepatitis B carriers were usually in the immune tolerant phase for up to several decades. During this phase, the rate of spontaneous or treatment-induced HBeAg seroconversion was less than 5% per year, and the range of fluctuating HBV DNA level was less than 1log_10_ IU/mL [26–30]. This suggests that the rate of spontaneously virological response within 52 weeks was no more than 5% in chronic hepatitis B carriers without effective treatment. Based on prior experience with TCM in treating chronic hepatitis B carrier, we assumed the occurrence rate of virological response within 52 weeks is 17%. To detect a difference in virological response between the treatment group and the control group with 80% power and a 5% level of significance, the sample size required per group was 103 according to the formula *n* = [(*Zα*/2+*Zβ*/2)^2^ × {*p*1(1 − *p*1) + *p*2(1 − *p*2)}]/(*p*1 − *p*2)^2^ [31]. With the randomization weighted 2 : 1 and allowing a patient dropout rate of approximately 20%, it is estimated that 200 patients in the treatment group and 100 patients in the control group would be enough for this study based on conservative principles.

### 2.7. Statistical Analysis

The intention-to-treat analysis included all patients who were randomly allocated to one of the two groups. A last observation carried forward analysis was conducted for any missing data of primary or secondary outcomes. Analysis of safety included data for all patients who had taken at least one dose of study medication after randomization. SPSS 15.0 package (SPSS Inc., Chicago, IL, USA) was used to perform the analysis. Continuous variables were expressed as mean ± standard deviation. An independent samples *t*-test was performed to calculate differences between prior and after treatment in one group. A paired samples *t*-test was used to compare difference between the two groups. Categorical variables were expressed as absolute and relative frequencies. The Chi-square test or Fisher exact test was used to compare the difference in proportions between the two groups. A *P* value < 0.05 at a two-tailed test was considered statistically significant.

## 3. Results

### 3.1. Demographic Data and Baseline Characteristics

The study was conducted between May 2009 and May 2011. A total of 2837 patients with a confirmed diagnosis of chronic hepatitis B carrier were screened. Among them, 372 patients met the inclusion criteria except liver histology then underwent liver biopsy. Finally 300 patients with only mild inflammatory necrosis and fibrosis, without bridging or confluent necrosis, were enrolled and randomized to receive either Chinese Herbal Formula (200 patients) or placebo (100 patients) treatment. The baseline demographic and clinical characteristics of the two groups were similar and summarized in Table 3. There was no significant difference in the age, gender ratio, ALT levels, and virological baseline characteristics between the two cohorts. 

**Figure 1:**
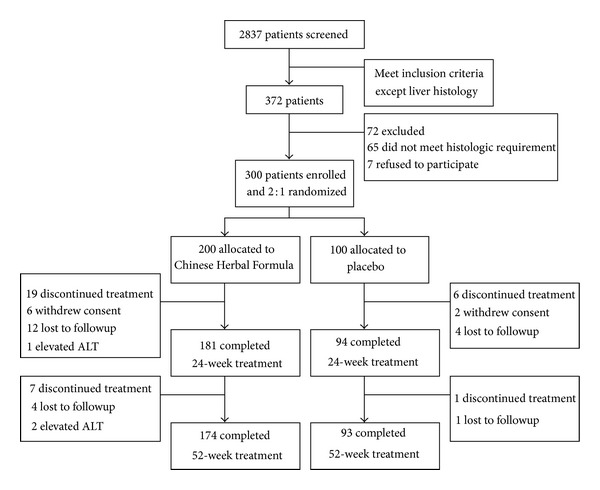
Study flow chart.

Review of the patient charts indicated that most of recruited patients had good compliance during treatment. However, at week 24, 25 patients were lost, including 19 who were treated with Chinese Herbal Formula and 6 in the control group. At week 52, another 8 patients were lost, including 7 in the treatment group and 1 in the control group. The common reason for discontinuing treatment was withdrawal of consent and lost to followup (details shown in Figure 1). Thus, 174 (87%) patients in the treatment group and 93 (93%) patients in the control group completed the 52-week treatment (Figure 1). 

### 3.2. Virological Response

The proportion of patients with reduced HBV DNA levels of >2log_10_ IU/mL were 7% (14/200) at week 24 and 19% (38/200) at week 52 for the treatment group, as compared to 3% (3/100, *P* = 0.1578) and 5% (5/100, *P* = 0.0011), respectively, for the control group. Among patients in the treatment group, serum HBV DNA was undetectable in 0.5% (1/200) at week 24 and 0.5% (1/200) at week 52; in the control group it was in 0% (0/100) and 1% (1/100), respectively. There was no significant difference between the two groups (*P* = 1.0000 and *P* = 1.0000, resp.) (Table 4).

At week 24, the mean reductions from baseline in serum HBV DNA level in the treatment and the control groups were 0.34log_10_ and 0.06log_10_ IU/mL, respectively (*P* = 0.0015 between the two groups). When categorized by baseline HBV DNA levels, the mean reductions in HBV DNA levels in each of the groups was 0.48log_10_ (treatment) and 0.05log_10_ IU/mL (placebo), respectively, for baseline levels in the range of 5 to <7log_10_ IU/mL (*P* = 0.0584). For patients with baseline HBV DNA values ranging from 7 to <9log_10_ IU/mL, the corresponding decreases were 0.26log_10_ and 0.06log_10_ IU/mL, respectively (*P* = 0.0822). For patients with baseline HBV DNA values >9log_10_ IU/mL, the corresponding decreases were 0.63log_10_ and 0.05log_10_ IU/mL, respectively (*P* = 0.0016). Similarly, at week 52, the mean reductions from baseline HBV DNA levels in serum in the treatment group and the control groups was 0.86log_10_ and 0.05log_10_ IU/mL, respectively (*P* = 0.0000). When categorized by baseline HBV DNA levels, the mean reduction in HBV DNA levels in each of the groups was 1.10log_10_ (treatment) and 0.05log_10_ IU/mL (placebo), respectively, for baseline levels in the range of 5 to <7log_10_ IU/mL (*P* = 0.0002). For patients with baseline HBV DNA values ranging from 7 to <9log_10_ IU/mL, the corresponding decreases were 0.76log_10_ and 0.05log_10_ IU/mL, respectively (*P* = 0.0000). For patients with baseline HBV DNA values >9log_10_ IU/mL, the corresponding decreases were 1.05log_10_ and 0.06log_10_ IU/mL, respectively (*P* = 0.0005) (Table 4). At the end of the treatment period (week 52), 13% (26/200) of treated patients and 3% (3/100) of patients given placebo had HBV DNA levels decrease to <5log_10_ IU/mL (*P* = 0.0057).

### 3.3. Serological Response

At week 24, there was no obvious difference between the two groups in the rate of patients who achieved HBeAg loss (3.5% (7/200) versus 2.0% (2/100), *P* = 1.0000), as well as HBeAg seroconversion (3.0% (6/200) versus 1.0% (1/100), *P* = 0.4989). After 52 weeks of treatment, when HBeAg loss was compared in treatment and control groups (5.5% (11/200) versus 3.0% (3/100), resp.), no differences were seen (*P* = 0.9135). Analogous results were obtained when assaying for HBeAg seroconversion (4.0% (8/200) versus 3.0% (3/100), *P* = 0.4981).

**Figure 2:**
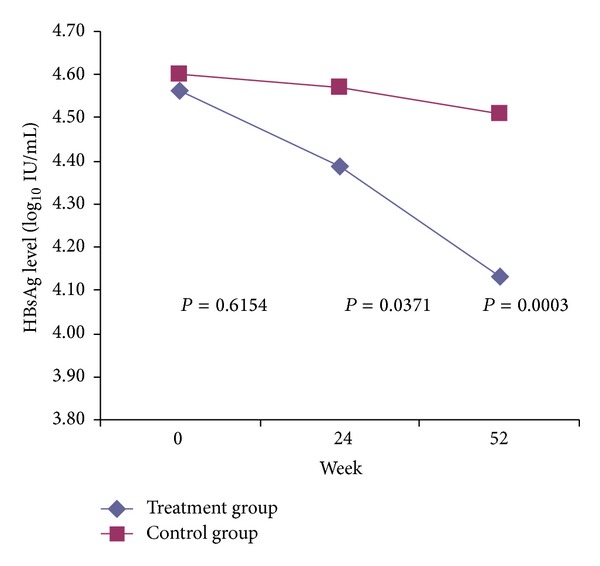
Concentration curves of HBsAg level during Chinese Herbal Formula and placebo treatment. There was no significant difference between the two groups in serum HBsAg level at baseline (*P* = 0.6154). The patients in the treatment group showed a significantly decreased HBsAg level in serum compared with control group at week 24 (*P* = 0.0371) and week 52 (*P* = 0.0003). Treatment group, Chinese Herbal Formula; Control group, placebo.

HBsAg levels declined from the pretreatment levels of 4.56 (±0.62) to 4.39 (±0.73) by week 24, and to 4.13 (±0.87) log_10_ IU/mL by week 52 of CHF treatment (*P* = 0.0125, *P* = 0.0000, resp.). Among placebo treated patients, HBsAg levels were 4.60 (±0.60) prior to treatment, 4.57 (±0.67) at week 24, and 4.51 (±0.75) log_10_ IU/mL at week 52 (*P* = 0.7391, *P* = 0.3499, resp.) (Figure 2). Taking a change of ≥0.5log_10_ IU/mL as significant, only 23 (11.5%) of the 200 patients in the treatment group and 4 (4%) of the 100 patients in the control group showed a significant decline in HBsAg levels at 24 week (*P* = 0.0324). Whereas at the end of 52-week treatment, a higher rate of significant decline in HBsAg levels was observed in the treatment group compared to the control group (27% (54/200) versus 7% (7/100), resp., *P* = 0.0000).

### 3.4. Serum Cytokine Levels

**Figure 3:**
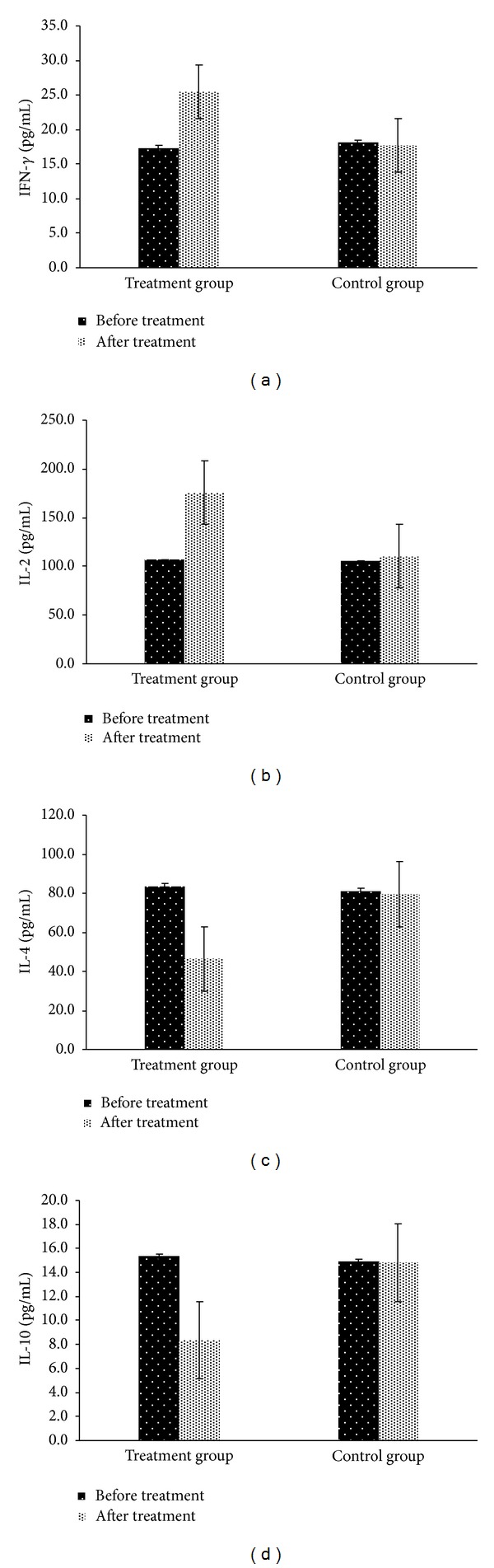
Serum levels of IL-2, IL-4, IL-10, and IFN-*γ* were determined by ELISA. The patients in treatment group showed significantly increased IL-2 (a) and IFN-*γ* (b) levels in serum compared with placebo group, as well as before treatment, *P* < 0.01, respectively. The patients in treatment group showed a significantly decreased IL-4 (c) and IL-10 (d) levels in serum compared with placebo group, as well as before treatment, *P* < 0.01, respectively. Treatment group, Chinese Herb Formula; control group, placebo.

After 52 weeks of CHF treatment, patients showed a significant increase in the mean levels of serum IFN-*γ* and IL-2 compared to the levels of these cytokines determined prior to treatment (25.51 ± 12.28 versus 17.34 ± 13.63, *P* = 0.0000; 175.69 ± 99.69 versus 106.70 ± 69.11, *P* = 0.0000, resp.). In addition, there was a marked decrease in the mean serum levels of IL-4, and IL-10 (46.45 ± 28.64 versus 83.78 ± 53.85, *P* = 0.0000; 8.36 ± 5.26 versus 15.33 ± 7.31, *P* = 0.0000, resp.). In contrast, there were no differences in levels of serum IFN-*γ*, IL-2, IL-4 and IL-10 before and after treatment with placebo in the control group (18.14 ± 11.94 versus 17.75 ± 7.73, *P* = 0.786; 105.58 ± 65.69 versus 110.43 ± 54.92, *P* = 0.572; 80.84 ± 42.88 versus 79.53 ± 60.36, *P* = 0.859; 14.92 ± 6.06 versus 14.84 ± 7.97, *P* = 0.943; resp.). Further, there were higher IFN-*γ* and IL-2 levels as well as lower IL-6 and IL-10 levels in the CHF treated group compared to patients administered placebo (*P* = 0.0000, resp.) (Figure 3).

### 3.5. Adverse Events

The Chinese Herbal Formula was well tolerated. The incidence of adverse events in the CHF treated group was similar to that in the control (placebo) group, with the exception that the CHF treated patients had an increased appetite (17% versus 1%, *P* = 0.0000). The majority of adverse events were not verified to be related to drug. The most often occurred drug-related adverse events were gastrointestinal symptoms such as abdominal pain, abdominal distension, diarrhea, stomach disturbances, and appetite decreases. With reference to these most common adverse events, the treatment group had many more patients with abdominal pain (4.5% versus 3%, *P* = 0.5320), abdominal distension (7.5% versus 2%, *P* = 0.0521), diarrhea (7% versus 3%, *P* = 0.1578), and stomach disturbances (9.5% versus 5%, *P* = 0.1756) than those of the placebo group, but there were no statistical significant differences between the two groups. Adverse events were generally transient and mild to moderate, which were self-limited or well responded to conventional treatment. Three patients discontinued treatment in the CHF group because of the elevated ALT. Of them, one patient experienced increased serum ALT (<2 × ULN, upper limit of normal) at week 24, and it resolved spontaneously after the study drug was stopped. Another 2 patients with elevated serum ALT were observed at week 36, whose ALT were both >5 × ULN (375 U/L and 287 U/L, resp.). The study drug was immediately stopped and Entecavir 0.5 mg daily was given. Serum ALT in these patients gradually returned to normal on subsequent visits. There were no other clinical laboratory abnormalities in the two groups, and no serious adverse events were observed (Table 5).

## 4. Discussion

Despite recent developments in the treatment of chronic hepatitis B (CHB), consisting of multiple nucleoside/nucleotide analogs, none are suitable to treat chronic HBV carriers during the immune tolerance phase of infection. The majority of these patients need an effective and safe antiviral therapy in order to decrease persistently high levels of HBV DNA and prevent progression to cirrhosis and HCC. Virological response was defined as undetectable serum HBV DNA or below detection limit (complete virological response) or reductions in serum HBV DNA levels of ≥2log_10_ IU/mL (partial virological response) [12]. In this study, compared with placebo, the Chinese Herbal Formula significantly increased the proportion of patients with reductions in serum HBV DNA of ≥2log_10_ IU/mL from baseline, whereas it does not enhance the rate of patients with undetectable serum HBV DNA level after 52 weeks of treatment. Meanwhile, patients undergoing CHF therapy had a greater mean reduction in serum HBV DNA from pretreatment values compared to the control group, in which no significant reduction was observed. Among patients with pretreatment HBV DNA levels between 5 and 7log_10_, CHF treatment resulted in a mean reduction in HBV DNA of 1.10log_10_ IU/mL. These results suggest that CHF is effective in inhibiting virus replication in chronic HBV carriers, but it is not potent enough to facilitate HBV DNA clearance over 52 weeks.

HBeAg seroconversion, involving the clearance of HBeAg and the appearance of anti-HBe, is considered a marker of durable remission and an appropriate end point of anti-HBV therapy in HBeAg-positive patients with CHB [32, 33]. In the current study, there were no obvious differences in HBeAg loss and HBeAg seroconversion between the treatment and control groups at week 52. However, since HBeAg seroconversion is also associated with a sustained reduction in HBV-DNA levels and always occurred in patients with HBV-DNA concentrations reduced to <3log_10_ copies/mL [34, 35], perhaps this result may change with prolonged treatment. Interestingly, the present study found that treatment with CHF was associated with a dramatic overall decline in HBsAg levels from pretreatment levels. Simultaneously, a higher percentage of patients with decline of ≥0.5log_10_ IU/mL from baseline was observed in the CHF treated group (27%, 54/200) compared with the control group (7%, 7/100). These effects on HBsAg levels are of particular importance because a recent study has confirmed that an early decline of 0.5log_10_ was a significant predictor of sustained virological response [36]. This decline in HBsAg levels may signify an immunological response that is independent of HBV DNA suppression during antiviral therapy, based on its positive correlation with intrahepatic HBV covalently closed circular (ccc) DNA, the main replicative template of HBV [37, 38]. 

It is well known that HBV clearance is mainly mediated by antiviral host immune responses. IFN-*γ* and IL-2 secreted by Th1 cells, which can enhance the human cellular immune response and benefit virus clearance, are principally involved in cell-mediated immunity. IL-4 and IL-10 have been reported to be higher in CHB and are secreted by Th2 cells which play a negative role in regulating the immune response and may be associated with the immune tolerant state of chronic HBV infection [39–41]. In the present study, there was significant upregulation of IFN-*γ* and IL-2 levels and reduction of IL-6 and IL-10 levels after 52 weeks of treatment compared to before therapy in the CHF treated compared to the control group. It is plausible that the host immunoreaction can be modulated by the administration of CHF, with a view to the medication's effective action against HBV.

Most often, chronic HBV carrier state is asymptomatic. In ancient China, there was no description for such a disease. For the past few years, we raised a theory called “Kidney asthenia and hepatic blood prostrated by dampness-heat” as the pathogenesis of chronic HBV carrier state based on the natural history and clinical manifestation of chronic HBV infection and “Seasonal Febris Theory” of Traditional Chinese medicine [42]. In the course of infection, dampness heat was considered as the initiating agent. Spleen-kidney asthenia was its underlying factor; stagnation of the liver meridian was a critical section of its pathology. Simultaneous presentation of sthenia and asthenia acted upon each other, making the disease refractory. In this context, the main principles of treating chronic HBV infection are strengthening the spleen and invigorating the kidney, clearing away the heat evil and expelling superficial evils, activating blood circulation, and eliminating dampness.

Based on the above information and clinical experiences, we raised an empirical formula, the recipe of invigorating kidney, and strengthening spleen, in which Tu Si Zi, Xian Ling Pi, Du Zhong, and Huai Niu Xi can invigorate the kidney and be used as the principal medications. They can aim directly at the nature of kidney asthenia and expel the evils. According to the modern pharmacological studies, Xian Ling Pi was proved to improve immune function [43], Tu Shi Zhi has been found to possess various pharmacological functions including improving immune and protecting hepatic mitochondria as well as preventing hepatic injury [44, 45]. Ye Xia Zhu has known strong anti-HBV activities [16–18], which is bitter and cold and can clear away the heat evil and expel superficial evils. Huang Qi, Bai Shu, Fu Ling, and Zhu Ling can invigorate spleen and eliminate dampness and be used as ministerial medications. Zhi Ke, Dan Shen, San Qi, and Yu Jin can promote Qi and activate blood circulation to dissipate blood stasis and be used as adjuvants. Gou Qi Zi as a messenger can lead other medications to hepatic blood. Thus, the whole recipe aims at the core pathogenesis of chronic HBV carrier state, in which monarch, minister, assistant, and guide are reasonably put in place. Both spleen and kidney are invigorated while both asthenia and sthenia are treated. This approach integrates three therapeutical principles: clearing, invigorating, and promoting. Overall, it can strengthen spleen and invigorate kidney, clear away heat evil and expel superficial evils, promote blood circulation, and eliminate dampness [46]. 

Treatment with CHF was generally safe and well tolerated in this study. A number of patients experienced transient and not severe adverse effects in the course of treatment, and most of complaints reported were similar between the CHF and control groups. The most common adverse events were gastrointestinal symptoms, which were probably related to use of oral dissolved medicines for a long time because some components of them, when fasting, will stimulate the gastric mucosa. In consideration of some patients usually suffering from these symptoms under fasting condition, we recommend that the Chinese Herbal Formula should be taken 2 hours after a meal. Actually, the patients experienced less discomfort in the abdominal area since following this principle. Increased appetite associated with CHF should be beneficial to helping patients invigorate health effectively. This can be interpreted by theory of Traditional Chinese Medicine which states that “strengthening spleen is able to promote digestive absorption of nutriment.” Three patients treated with CHF were withdrawn because serum ALT increases were observed. However, the one with ALT <2 × ULN recovered spontaneously after stopping treatment. Another 2 patients with ALT >5 × ULN eventually resolved after appropriate treatment. Spontaneous elevations of ALT, or flares, are a well-recognized phenomenon in patients treated with PEG-IFN [47]. Two different types of flares were recognized during PEG-IFN therapy of HBeAg-positive chronic hepatitis B. Host-induced flares, characterized by an ALT flare followed by a subsequent decrease in HBV DNA, were associated with response to treatment and may result in decline and clearance of HBV DNA, HBeAg, and HBsAg. In contrast, virus induced flares, which occurred after an increase in HBV DNA level and most probably was indicative for increased expression of viral antigens, did not lead to treatment response [48, 49]. In the current study, with respect to reduction of HBV DNA level simultaneously occurred in these two patients with elevated ALT, which conform to the character of host induced flare, we speculated elevated ALT was due to the immune clearance. Furthermore, our results suggested the CHF had the positive effect on immune function. Further investigations are required to determine whether long-term the treatment leads to significant increase in serum ALT, which is one of the strongest signs of a chronic hepatitis B carrier entering to immune clearance phase. 

To our knowledge, this is the first time that the efficacy and safety of Chinese Herbal Formula on chronic hepatitis B carriers has been evaluated in a prospective, multicenter double-blind, random placebo-controlled, and large-scale registration trial. In this work, the therapeutic effect indexes incorporated quantification of HBV DNA, HBsAg, and HBeAg determined by the most accurate methods at present, which provided a comprehensive understanding of Chinese Herbal Formula in inhibiting HBV replication. Although the results of the present study should be persuasive, there are some potential limitations in this study. First, all of the patients were ethnic Han Chinese adults from China. Applicability of these outcomes to other regions and ethnicities, therefore, is uncertain. Second, because of restrictions on time and funding, the follow-up analysis of the sustained effect of CHF has not been evaluated. These data could be the starting point for future research to understand long-term impact of the Chinese Herbal Formula on chronic HBV carriers.

Given the lack of recommended treatment options for chronic hepatitis B carrier who is still in the risk of progression to cirrhosis and HCC, any complementary and alternative medication that shows a certain degree of antiviral effect should not be ignored. In short future, a longer, randomized controlled trial of the Chinese Herbal Formula may be helpful to determine whether it would get better curative effect against HBV and keep sustained virus response, and even change the long-term prognosis of chronic HBV carriers, by increasing the duration of treatment and a consecutive long-term followup. 

## 5. Conclusions

The Chinese Herbal Formula (invigorating kidney and strengthening spleen) is safe and effective in inhibiting HBV replication in chronic hepatitis B virus carriers. The ability of the compound to modulate host immune function probably contributed to this effect.

## Figures and Tables

**Table 1:** Inclusion and exclusion criteria of patients.

Inclusion criteria	Exclusion criteria
Conform with the diagnostic criteria of chronic hepatitis B carriers	Inactive HBsAg (+) carriers
Conform with the pathogenesis and syndromes of kidney deficiency and spleen deficiency	Serum *α*-fetoprotein abnormal
Age 20–65 y	Pregnancy or breast feeding
ALT ≤ 40 IU/L	Coinfection with HIV, HCV, HDV
HBV DNA > 100,000 IU/mL	Histologic evidence of cirrhosis
Liver biopsy before treatment indicates mild inflammatory necrosis and fibrosis	Evidence of any other chronic liver disease
Sign informed consent and participate in this clinical trial voluntarily	Mental illness or any other serious systemic illness Antivirus treatment with nucleoside or interferon-*α* within 6 months Abuse alcohol or illegal drugs Allergic to the drug ingredients

**Table 2:** The list of raw herbs composing the Chinese Herbal Formula.

Chinese name	Full scientific name	Parts of plant use	Dose of dry plant (grams)	Dose after extraction (grams)
Xian Ling Pi	Epimedium brevicornum Maxim	Overground part	30	1.5
Tu Si Zi	*Cuscuta chinensis* Lam.	Mature seed	10	0.5
Du Zhong	*Eucommia ulmoides* Oliver	Plant bark	15	0.75
Huai Niu Xi	*Achyranthes bidentata* Bl.	Root	15	6
Ye Xia Zhu	*Phyllanthus urinaria* Linn.	Whole plant	15	6
Huang Qi	*Astragalus membranaceus* (Fisch.) Bge.	Root	15	1.5
Bai Shu	*Atractylodes macrocephala* koidz	Rhizome	15	2.55
Fu Ling	*Poria cocos* (Schw.) Wolf	Sclerotium	15	1.5
Zhu Ling	Polyporus umbellatus (Pers.) Fries	Sclerotium	10	1.0
Zhi Ke	Fructus Aurantii	Immature fruits (pulp removed)	15	2.55
Dan Shen	*Salvia miltiorrhiza* Bge.	Root and rhizome	20	3
San Qi	*Panax notoginseng* (Burk.) F. H. Chen	Root	5	5
Yu Jin	Radix curcumae	Tuberous root	15	0.75
Gou Qi Zi	*Lycium barbarum* L.	Mature fruit	15	6

Total			210	38.6

**Table 3:** Baseline characteristics of the total study patients.

Variable	Treatment group (*n* = 200)	Control group (*n* = 100)	*χ* ^2^/*t*	*P* values
Age, mean (SD), y	33.34 (6.86)	34.01 (8.32)	0.7415	0.4590^#^
Range	21–59	22–63		
Sex, *n* (%)			0.1904	0.6625^*※*^
Male	137 (68.50)	66 (66.00)		
Female	63 (31.50)	34 (34.00)		
Clinical course, mean (SD), m	86.35 (72.13)	90.56 (70.48)	0.4808	0.6310^#^
ALT, mean (SD), U/L	26.40 (8.71)	26.57 (7.92)	0.1640	0.8698^#^
HBV DNA, mean (SD), log_10_ IU/mL	8.15 (1.13)	8.17 (1.02)	0.1479	0.8825^#^
HBV DNA baseline level, *n* (%)				
5 to <7 log_10_ IU/mL	28 (14.00)	11 (11.00)	0.5305	0.4664^*※*^
7 to <9 log_10_ IU/mL	141 (70.50)	74 (74.00)	0.4022	0.5260^*※*^
>9 log_10_ IU/mL	31 (15.50)	15 (15.00)	0.0128	0.9098^*※*^
HBsAg, mean (SD), log_10_ IU/mL	4.56 (0.62)	4.60 (0.60)	0.5030	0.6154^#^

SD: standard deviation; ALT: alanine aminotransferase; HBV: hepatitis B virus; HBsAg: hepatitis B surface antigen.

^*※*^Chi-square test.

^#^ *t*-test.

**Table 4:** Virological response and change of serum HBV DNA level after treatment.

Treatment response	Treatment group (*n* = 200)	Control group (*n* = 100)	*χ* ^2^/*t*	*P* values
24 weeks				
Patients with HBV DNA level decline >2 log_10_ IU/mL, *n* (%)	14 (7.00)	3 (3.00)	1.9954	0.1578^*※*^
Patients with undetectable HBV, *n* (%)	1 (0.5)	0 (0)	0.0000	1.0000^*※*★^
Reduction in HBV DNA level, mean (SD), log_10_ IU/mL				
Total	0.34 (0.92)	0.06 (0.62)	3.2094	0.0015^△^
baseline level 5 to <7 log_10_ IU/mL	0.48 (0.98)	0.05 (0.39)	1.9532	0.0584^△^
baseline level 7 to <9 log_10_ IU/mL	0.26 (0.96)	0.06 (0.67)	1.7470	0.0822^△^
baseline level >9 log_10_ IU/mL	0.63 (0.58)	0.05 (0.46)	3.3742	0.0016^#^
52 weeks				
Patients with HBV DNA level decline >2 log_10_ IU/mL, *n* (%)	38 (19.00)	5 (5.00)	10.6416	0.0011^*※*^
Patients with undetectable HBV^ a^, *n* (%)	1 (0.5)	1 (1.00)	0.0000	1.0000^*※*★^
Reduction in HBV DNA level, mean (SD), log_10_ IU/mL				
Total	0.86 (1.27)	0.05 (0.86)	6.4772	0.0000^△^
baseline level 5 to <7 log_10_ IU/mL	1.10 (0.79)	0.05 (0.47)	4.0849	0.0002^#^
baseline level 7 to <9 log_10_ IU/mL	0.76 (1.38)	0.05 (0.47)	4.4885	0.0000^△^
baseline level >9 log_10_ IU/mL	1.05 (1.06)	0.06 (0.68)	3.8132	0.0005^△^

SD: standard deviation; HBV: hepatitis B virus.

^a^HBV DNA < 12 IU/mL.

^*※*^Chi-square test.

^#^ *t*-test.

^△^Adjusted *t*-test.

^★^Continuity correction.

**Table 5:** Adverse events in patients reported in the two groups during treatment.

Variable	Treatment group Number of patients (%) (*n* = 200)	Control group Number of patients (%) (*n* = 100)	*χ* ^ 2^ (Chi-square test)	*P* values
Abdominal pain	9 (4.5)	3 (3)	0.3906	0.5320
Abdominal distension	15 (7.5)	2 (2)	3.7726	0.0521
Diarrhea	14 (7)	3 (3)	1.9954	0.1578
Nausea	5 (2.5)	2 (2)	0.0000^△^	1.0000
Increased appetite	34 (17)	1 (1)	16.5606	0.0000
Appetite decreases	5 (2.5)	4 (4)	0.1289^△^	0.7196
Feel thirsty	4 (2)	3 (3)	0.0183^△^	0.8924
Stomach disturbances	19 (9.5)	5 (5)	1.8342	0.1756
Constipation	4 (2)	3 (3)	0.0183^△^	0.8924
Malaise	5 (2.5)	2 (2)	0.0000^△^	1.0000
Insomnia	3 (1.5)	1 (1)	0.0000^△^	1.0000
Headache	3 (1.5)	2 (2)	0.0000^△^	1.0000
Dizziness	5 (2.5)	1 (1)	0.1913^△^	0.6618
Fatigue	3 (1.5)	2 (2)	0.0000^△^	1.0000
Chest tightness	4 (2)	3 (3)	0.0183^△^	0.8924
URTI syndromes^*※*^	3 (1.5)	3 (3)	0.1913^△^	0.6618
Mouth ulceration	3 (1.5)	2 (2)	0.0000^△^	1.0000
Rash	5 (2.5)	0 (0)	1.2458^△^	0.2644
Dysuria	2 (1)	0 (0)	0.0629^△^	0.8019
Urethral burning pain	2 (1)	0 (0)	0.0629^△^	0.8019
Increased serum ALT^*※*^	3 (1.5)	0 (0)	0.3788^△^	0.5383

^*※*^URTI: upper respiratory tract infection; increased serum ALT defined as >40 U/L.

^△^Correction.
